# Prevalence and risk factors for khat use among youth students in Ethiopia: systematic review and meta-analysis, 2018

**DOI:** 10.1186/s12991-020-00265-8

**Published:** 2020-03-09

**Authors:** Wondale Getinet Alemu, Tadele Amare Zeleke, Wubet Worku Takele, Shegaye Shumet Mekonnen

**Affiliations:** 1 Department of Psychiatry, College of Medicine and Health Science, University of Gondar, Gondar, Ethiopia; 2 Department of Community Health Nursing, School of Nursing, College of Medicine and Health Sciences, University of Gondar, Gondar, Ethiopia

**Keywords:** Khat chewing, Khat use, Students, Ethiopia

## Abstract

**Background:**

Khat use is a widely spreading public health problem affecting the most economically productive population areas in Ethiopia. Khat use among students has been linked with mental, physical, social, and psychological problems. Reliable prevalence has not been recognized because of varying published rates. The objective of this systematic review and meta-analysis is to synthesize evidence on the prevalence and potential risk factors of khat use in Ethiopia.

**Methods:**

We found 284 studies of which 266 were removed due to duplication, irrelevant topics, and other reasons, respectively. All studies conducted in Ethiopia on khat chewing among students irrespective of time frame were included. Subsequently, 18 studies were used for synthesis of prevalence. Figures were extracted from published reports, and any lost information was requested from investigators. The quality of the included literature was evaluated by using the Newcastle–Ottawa Scale (NOS). Prevalence was pooled using random-effects meta-analyses. The presence of association was declared using P-values and an odds ratio with a corresponding 95% CI.

**Results:**

The pooled prevalence of khat use among students was 16.7% (13.7–19.7%). In the subgroup analysis, the highest prevalence was observed in the Oromia region, at 21.1% (15.5%, 26.7%), and an almost equal prevalence of 14.8% (10.6, 18.9) and 14.3% (10.3, 18.3) was observed in Amhara and the Southern Nation, Nationalities, and People’s Region of Ethiopia, respectively. Being male (OR: 2.43 (1.73, 3.13)), being a Muslim religion follower (OR: 2.22 (1.6, 2.8)), being an alcohol user (OR: 2.3 (1.5, 3.0)), khat use by a family member (OR: 1.8 (1.4, 2.2)), peer pressure (OR: 4.4 (3.1, 5.6)), and being a cigarette smoker (OR: 8.5 (5.3, 11.7)) were found to be risk factors for khat chewing.

**Conclusions:**

Khat use is a common problem among students. Health promotion, awareness on effect of khat, set policy on khat and substance use on the male sex, Muslim religion, alcohol user, having a family-member khat user, peer pressure, and being a cigarette smoker as possible risk factors for khat use among students.

**Limitations:**

Irrespective of time restriction, all studies conducted in Ethiopia are included and cross-sectional in nature. Protocol no. CRD-42017081886.

## Background

Khat is a plant containing a natural psychoactive substance which is cultivated in East African as well as Arab lands [1]. Khat has different names in different countries, but “khat” remains the name widely used in studies [2]. The origin of khat is not known, but it is believed to be native to Ethiopia and was originally used there [3]. Khat contains the amphetamine-like substances cathine, cathinone, and methcathinone [4].

Khat has a stimulant effect on the body [5, 6]. The fresh green leaves and young buds are chewed [7]. This stimulates both the peripheral and vital nervous system, causing, for instance, insomnia, alertness, anorexia, and increased respiration, body temperature, blood pressure, and heart rate [8]. The stimulant effect is mutually enhanced by caffeine use and cigarette smoking [9]. Khat use has appeared to be a male habit, but women practise it as well [10]. Users start chewing at an early age and develop an uncontrollable habit lasting throughout the lifespan [11]. It is practised based on local customs and traditions [12] and carried out in religious ceremonies [13, 14].

The World Health Organization report has shown that khat use causes dependency [15–19], predisposes the individual to myocardial infarction [20], ischemic heart disease [21], psychosis [22, 23], distress [24], premature ejaculation [25], unprotected sex [26], manic episodes [27, 28], oesophageal cancer [29], low birth weight and lactation problems [30], structural and functional brain changes [31, 32], and criminal activity [33].

Khat chewing is a familiar habit among students for staying alert, achieving higher concentration at work, socializing, and providing relaxation, relief from stress, and a desire to study for long hours [34–37].

Moreover, being male, having chewer friend(s), believing that chewing khat will boost performance, drinking alcohol, and having a family that cultivates khat were found to considerably increase the chewing practice [34, 38–40].

The literature includes studies conducted in Ethiopia among students. However, the literature shows a difference in prevalence and associated factors. Therefore, this systematic review and meta-analysis aim to estimate the pooled prevalence and associated factors of current use of khat chewing among students in Ethiopia.

## Methods

PubMed/MEDLINE, Scopus, HINARI and EMBASE were searched for published studies. In addition, 10 pages were accessed using Google Scholar. All references in the relevant articles were reviewed in order to obtain other studies. Furthermore, for partial articles or those missing necessary information, the authors of the articles were contacted via email or other means of communication. For the PubMed search, the following terms were applied: ‘khat chewing’, ‘khat use’, ‘chewing habit’, ‘determinant factors*’, ‘student’, ‘college students’ and ‘Ethiopia’. An advanced search was conducted using these terms with the options ‘MeSH terms’ and ‘all fields’ selected and including ‘AND’ and ‘OR’ Boolean operators as appropriate. The rest of the electronic databases were searched using database-specific subject headings linked with the terms and keywords used in PubMed. “Preferred Reporting Items for Systematic Reviews and Meta-Analyses (PRISMA)” guidelines used [41]. To show the procedures used for the screening and selection processes, a PRISMA flow diagram was used. The findings of this meta-analysis are presented here in with the aid of figures.

### Review and meta-analysis registration

This systematic review and meta-analysis were registered at the International Prospective Register of Systematic Reviews. The following represents the registration number: CRD-42017081886.

### Eligibility criteria

Three investigators (WG, TA and WW) independently screened the selected articles using their titles and abstracts before retrieving the full-text papers. We use pre-specified inclusion criteria to screen the full-text articles. Disagreements between the investigators were discussed during a consensus meeting with a fourth reviewer (SS) in order to select the studies to be included in the systematic review and meta-analysis.

### Inclusion criteria

- Cross-sectional studies.
- Studies on khat chewing among students.
- Studies published in English.
- Studies reporting on the prevalence and/or determinants of khat chewing.
- Studies conducted in Ethiopia.

### Exclusion criteria

- Editorials, letters, reviews, commentaries and interventional studies.
- Studies without access to the full data even after contacting the author(s).
- Duplicate studies.

### Data extraction

All the articles accessed using the databases and search engines were exported to EndNote (version 6), and we excluded duplicate articles. The remaining articles were evaluated based on the topic, language and study area. Next, studies conducted outside of Ethiopia, those not published in English and those on irrelevant topics were excluded. There were no time restrictions among the included studies. Finally, the abstracts and full text of the remaining articles were reviewed.

### Outcome variable

Current khat use is defined as the proportion of students who are chewing for different purposes within 3 months of prior to data collection.

### Data synthesis and quality assessment

After extracting and documenting the data in a Microsoft Excel spreadsheet, we exported it to Stata (version 14) for further analysis. All the analyses were conducted using Comprehensive Meta analysis software (version 3) [42]. The overall pooled prevalence of khat chewing was estimated using a random-effects meta-analysis [43]. First, using a fixed-effects model, heterogeneity among the studies was determined. A Q test and an I^2^ heterogeneity test [43] were used to declare heterogeneity at *p* < 0.05. The prevalence of statistical heterogeneity between the studies was assessed using I^2^ statistics, with 25%, 50% and 75% representing low, medium and high heterogeneity, respectively [44]. The quality of the included studies was evaluated using the Newcastle–Ottawa Scale [45] and tested based on sample size and representativeness, comparability between participants, ascertainment of khat chewing and statistical quality. To test the agreement between the three reviewers, the actual agreement and agreement beyond chance (unweighted kappa) were used. The values of reviewers’ result 0, 0.01–0.20, 0.21–0.40, 0.41–0.60, 0.61–0.80, and 0.81–1.00 were used to represent poor, slight, fair, moderate, substantial, and almost perfect agreements, respectively [46]. A random-effects model was used in the analysis. Meta-regression was conducted to explore the probable source of heterogeneity. A leave-one-out sensitivity analysis was also conducted to assess which studies majorly impacted between-study heterogeneity. A funnel plot and Egger’s regression test were used to measure publication bias.

## Results

### Search outcomes

**Fig. 1:**
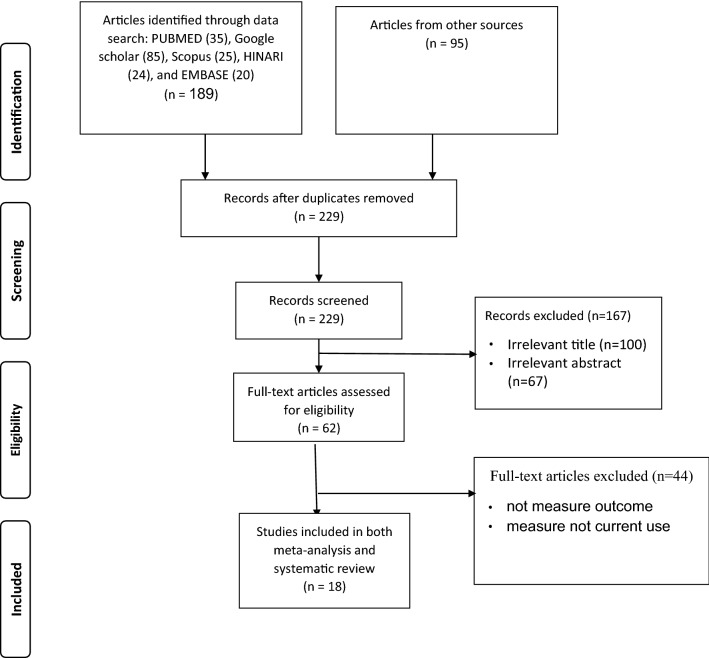
Flowchart describes the selection of studies for the systematic review and meta-analysis of prevalence and risk factors of khat chewing among students in Ethiopia, 2018

The systematic literature search generated a total of 284 articles. In total, 55 were duplicated and 167 were irrelevant, and, as such, these were excluded. In addition, 44 were excluded (not measure outcome, measure not current use). The remaining 18 articles were used to determine the pooled prevalence of khat chewing in Ethiopia. All 18 articles were cross-sectional studies (Fig. 1).

All the included studies were conducted in Ethiopia (Table 1). Finally, the levels of agreements between the reviewers about the levels of bias for studies included in this meta-analysis we got from moderate to almost perfect (Kappa statistic range 0.60–1) (Table 2).

**Table 1:** Descriptive summary of 18 studies reporting the prevalence and associated factors of khat chewing among students in Ethiopia included in the systematic review and meta-analysis, 2018

Author	Year	Region	Study area	Sample size	Response rate (%)	The objective of the study	Prevalence (95% CI)
Abdeta T	2017	Oromia	Jimma University	651	95.1	Prevalence, withdrawal symptoms and associated factors of khat chewing among students at Jimma University in Ethiopia	23.9 (14.50, 33.30)
Ayana A	2004	Oromia	Jimma University	500	97.1	Khat (Catha edulis Forsk) chewing, sociodemographic description and its effect on academic performance, Jimma University students	24.8 (15.28, 34.30)
Reda A	2012	Oromia	Harar Town	1890	91.1	Prevalence and determinants of khat (Catha edulis) chewing among high school students in eastern Ethiopia: a cross-sectional study	24.2(14.62, 33.78)
Dires E	2016	Oromia	Jimma town	296	100	Factors associated with khat chewing among high school Students in Jimma Town Southwest Ethiopia	14.2 (6.99, 21.41)
Astatkie A	2015	SNNPR	Hawassa University	1255	97.3	Prevalence of and factors associated with regular khat chewing among university students in Ethiopia	10.5 (4.18, 16.82)
Kassa A	2011	SNNPR	Hawassa University	590	94.5	Determinants of alcohol use and khat chewing among Hawassa University students, Ethiopia: a cross-sectional study	16.3 (8.50, 24.10)
Kassa A	2017	SNNPR	Sidama zone	1577	95.3	Prevalence of khat chewing and its effect on academic performance in Sidama zone, Southern Ethiopia	13.0(5.96, 20.04)
Kassa A	2014	SNNPR	Hawassa University	586	99.3	Prevalence and factors determining psychoactive substance (PAS) use among Hawassa University (HU) undergraduate students, Hawassa Ethiopia	20.3(11.62, 28.98)
Gebreslassie M	2013	Tigray	Axum University	764	98.7	Psychoactive substances use and associated factors among Axum University students, Axum Town, North Ethiopia	27.9 (17.74, 38.06)
Gebrehanna E	2014	Amhara	Bahir Dar University	3268	77.5	Khat chewing among Ethiopian University Students—a growing concern	12.7 (5.73, 19.67)
Teni FS	2015	Amhara	Gonder university	424	94.3	Prevalence, reasons, and perceived effects of khat chewing among students of a college in Gondar Town, Northwestern Ethiopia: a cross-sectional study	32.5 (21.76, 43.24)
Zein ZA	1998	Amhara	Gonder university	479	98.8	Polydrug abuse among Ethiopian university students with particular reference to khat (Catha edulis)	22.3(13.26, 31.34)
Bizuayehu G	2009	Amhara	Gonder	397	98.5	Prevalence, factors and consequences of khat chewing among high school students of Gondar Town, Northwestern Ethiopia	12.6(5.75, 19.45)
Aklilu S	2013	Amhara	Gonder university	302	97.4	Prevalence and associated factors of khat chewing among Atse Fasil Campus Students, University of Gondar, North West Ethiopia June	6.9 (1.84, 12.06)
Berihun AD	2015	Amhara	Gonder University	872	95.8	Khat use and Its determinants among University students in Northwest Ethiopia: a multivariable analysis	13.6(6.43, 20.77)
Lakew A	2014	Amhara	Ataye	332	88	Prevalence of Catha edulis (khat) chewing and Its associated factors among Ataye Secondary School Students in Northern Showa, Ethiopia	13.3 (6.26, 20.24)
Adere A	2017	Amhara	Woldia university	730	89.7	Determinants of psychoactive substances use among Woldia University students in Northeastern Ethiopia	10.4 (4.12, 16.68)
Yegazew K	2002	Amhara	Gonder university	1258	87.7	Cigarette smoking and khat chewing among college students in North West Ethiopia	17.5 (9.36, 25.64)

**Table 2:** The quality and agreed level of bias and level of agreement on the methodological qualities of included studies in a meta-analysis based on sampling, outcome, response rate and method of analysis

Study	Percentage of agreement	Kappa value	Level of agreement	Nos quality (score on 0 to 9 scale)
Abdeta T (2017)	100	1	Almost perfect	8
Ayana A (2004)	100	1	Almost perfect	9
Reda A (2012)	100	1	Almost perfect	8
Dires E (2016)	100	1	Almost perfect	8
Astatkie A (2015)	100	1	Almost perfect	8
Kassa A (2016)	100	1	Almost perfect	9
Kassa A (2017)	100	1	Almost perfect	9
Kassa A (2014)	100	1	Almost perfect	9
Gebreslassie M (2013)	100	1	Almost perfect	9
Gebrehanna E (2014)	100	1	Almost perfect	8
Teni FS (2015)	75	0.60	Moderate	7
Zein ZA (1998)	75	0.60	Moderate	7
Bizuayehu G (2009)	100	1	Almost perfect	9
Aklilu S (2013)	100	1	Almost perfect	8
Berihun AD (2015)	100	1	Almost perfect	9
Lakew A (2014)	100	1	Almost perfect	8
Adere A (2017)	100	1	Almost perfect	8
Yegazew K (2002)	100	1	Almost perfect	9

### Pooled prevalence of khat chewing among students

**Fig. 2:**
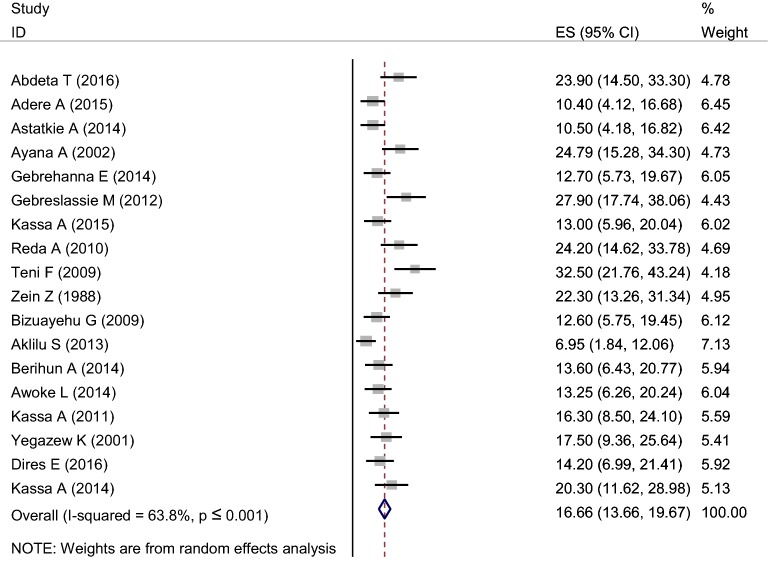
Forest plot of pooled prevalence of khat chewing in Ethiopian students 2018 (*n* = 18)

The pooled prevalence of khat chewing in Ethiopia was 16.7% (13.7–19.7: I^2^ = 63.8%, *p* ≤ 0.001) (Fig. 2).

**Fig. 3:**
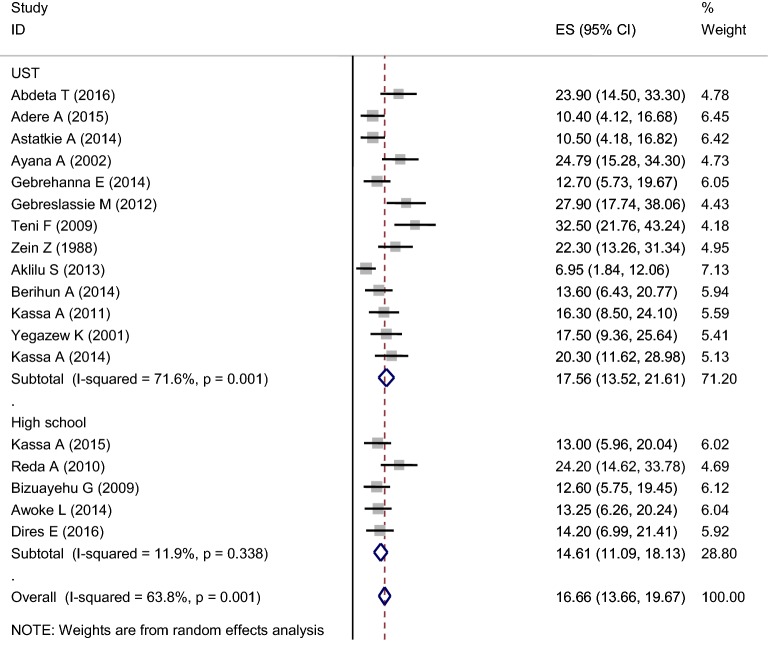
Subgroup analysis on prevalence of khat chewing among high school and university students 2018 (*n* = 18)

In the random-effects model, the subgroup analysis by setting on high school and university is different which is 14.61% (11.09, 18.13:I^2^ = 11.9%, *p* = 0.338) and 17.56% (13.52, 21.61:I^2^ = 71.6%, *p* = 0.001), respectively (Fig. 3).

**Fig. 4:**
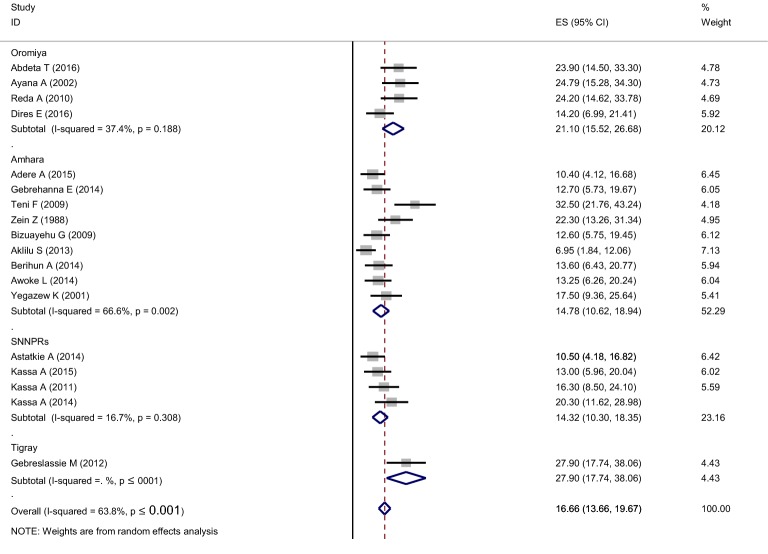
Subgroup prevalence of khat chewing in Ethiopian students, 2018 (*n* = 18)

By region showed that the highest prevalence of khat chewing was observed in the Oromia Region (OR: 21.1 (15.5, 26.7)). A comparable prevalence was observed in the Amhara Region (OR: 14.8 (10.6, 18.9)) and the southern Nation Nationality people of Ethiopia (OR: 14.3 (10.3, 18.3)) (Fig. 4).

### Publication bias

**Fig. 5:**
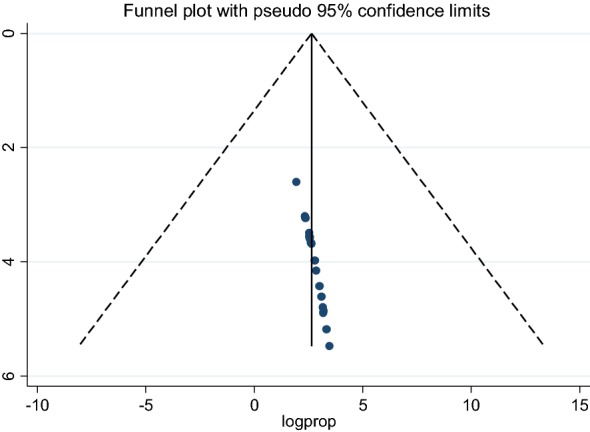
Funnel plot showing publication bias of prevalence studies among students, a systematic review and meta-analysis, Ethiopia, 2018

There was no evidence of bias, as observed in the funnel plot. An Egger’s regression test confirmed this (*p* = 0.53) (Fig. 5).

### Factors associated with khat use

Khat use is common among high school, college and university students. There are many risk factors, but just we include those reported in more than one study. Being male is more likely to chew khat than females (OR: 2.43 (1.73, 3.13). In addition, khat chewing is predominantly practised by Muslims (OR: 2.22 (1.6, 2.8). Students with a family member who had a history of khat use or who currently uses khat are more likely to chew khat than students without such a family member (OR: 1.8 (1.4, 2.2).

## Discussion

The objectives of this systematic review and meta-analysis were to assess the prevalence and associated factors of khat use among young students in Ethiopia. Young students who are habitual khat users believe that its use boosts alertness, concentration, imaginative abilities, and improves communication skills. To the best of our knowledge, this systematic review and meta-analysis is the first of its kind, assessing the pooled prevalence and factors that have an effect on the habitual use of khat among young students in Ethiopia. The overall pooled prevalence of khat use was found to be 16.7% (13.7–19.7). The pooled prevalence of Khat use was found to be different across regions; it was highest in the Oromia region, 21.10% (15.52, 26.68), and we found a similar prevalence in the Amhara and SNNPs regions, 14.78 (10.6, 18.9) and 14.3 (10.3, 18.3), respectively (figure-3). Clearly illustrated above, the subgroup analysis demonstrated that the pooled prevalence of khat use among young students is slightly different across different regions of Ethiopia. The possible reason for these variations could be environmental, religious, and/or cultural differences across the regions. For example, people residing in the Oromia region are Muslim followers. In addition to this, the higher prevalence of khat chewing in this region could be explained by the differences in settings across regions, such as access to khat and factors outside the university and high school environment.

A meta-analysis study previously completed on University students found that 23.22% (95% CI 19.5, 27.0%) of these students were chewers, which is a bit higher than our findings [43]. This discrepancy might be due to differences in study population, while ours considered young students on high school and university, the former one focused on university students, these students couldn’t afford to buy the khat and they might not use.

Multiple factors have contributed to young students to be khat users. Being male, younger age, religion, ethnicity, khat use by family, family history of other substances use, living condition, peer pressure, other psychoactive substance use, having a family that cultivates khat, perceive khat use boosts performance, increased class workload, residency, having suicidal ideation, having ever had a sexual contact were found to be the most important associated factors.

Gender of students continued to be a significant factor affecting students' behaviour. Being male sex had a significant role to be user as compared to females. This finding is line with studies conducted in Ethiopia [2, 36, 47–51]. This significant difference between male and female may be justified as; females are less exposed to chewing practice than males. Moreover, we found that being Muslim by religion had a significant role to be user than others [2, 36, 41, 43, 51, 52].

Having chewer friends was strong predictor of chewing which is similar to other studies [2, 41, 43, 45, 49, 50, 53], khat use by family member was associated with increased odds of use among participants. This finding was similar with studies conducted on substance abuse [2, 36, 48, 49]. The possible cause for the association may be because of shared influence and peer pressure.

Furthermore, in this review, it has been observed that other substance use is common among study participants. Who had ever drunk alcohol and ever smoked cigarette were more likely to practise chewing as compared to no alcohol and cigarette users. This finding is supported by r studies conducted, ever drunk alcohol [52, 54], and smoking cigarette [43, 48, 50, 52, 54]. These studies reported that history of alcohol consumptions and cigarette smoking was positively associated with chewing. This finding provides evidence on the prevalence of khat use on Ethiopian students with relevant data. Measures to reduce the use of khat should be taken and these should be considered as priority areas: awareness, family contribution, prevention and early intervention.

## Conclusions

Khat use is prevalent among high school, college and university students. The prevalence of khat use appeared to be high. Particular attention should be given to male gender, Muslims religion follower, alcohol users, having family member khat user, peer pressure, being cigarette smoker. There is a need for early intervention that targets high school, college, and university students to reduce the health, financial and social consequences of khat use.

## Limitations of the study

The limitation was that only English articles were considered to conduct this review. In addition, all of the studies included in this review were cross-sectional in nature, as a result the outcome variable might be affected by confounding variables.

## Data Availability

Not applicable.

## Abbreviations

AOR: Adjusted odds ratio
WHO: World Health Organization
COR: Crude odds ratio
CI: Confidence interval
OR: Odds
SPSS: Statistical Package for Social Science
MOOSE: Meta-analysis of observational studies
